# Associations between circulating adipokines and bone mineral density in patients with knee osteoarthritis: a cross-sectional study

**DOI:** 10.1186/s12891-018-1936-7

**Published:** 2018-01-17

**Authors:** Juan Wu, Jianhua Xu, Kang Wang, Qicui Zhu, Jingyu Cai, Jiale Ren, Shuang Zheng, Changhai Ding

**Affiliations:** 1 Department of Rheumatology and Immunology, Arthritis Research Institute, the First Affiliated Hospital of Anhui Medical University, 218 Jixi Street, Hefei, China; 2 Menzies Institute for Medical Research, University of Tasmania, Private Bag 23, Hobart, TAS 7000 Australia; 3 Institute of Bone & Joint Translational Research, Southern Medical University, Guangzhou, Guangdong China

**Keywords:** Adiponectin, Bone mineral density, Leptin, Osteoarthritis, Resistin

## Abstract

**Background:**

Associations between adipokines and bone mineral density (BMD) in knee osteoarthritis (OA) remain indistinct. The aim of this study was to investigate the cross-sectional associations between serum levels of adipokines and BMD in patients with knee OA.

**Methods:**

This study included 164 patients with symptomatic knee OA from the Anhui Osteoarthritis study. Serum levels of leptin, adiponectin, and resistin were measured using an enzyme-linked immunosorbent assay (ELISA). BMD at total body, spine, hip, and femur were measured by dual-energy X-ray absorptiometry (DXA).

**Results:**

In multivariable analyses, serum levels of leptin were significantly associated with reduced BMD at total body, hip, total femur, femoral neck, and femoral shaft (β = − 0.019, 95% CI -0.034 to − 0.005; β = − 0.018, 95% CI -0.034 to − 0.003; β = − 0.018, 95% CI -0.034 to − 0.002; β = − 0.016, 95% CI -0.032 to 0.000; β = − 0.026, 95% CI -0.046 to − 0.006; respectively). Serum levels of adiponectin were significantly and negatively associated with BMD at total femur and femoral shaft (β = − 0.007, 95% CI -0.013 to 0.000; β = − 0.011, 95% CI -0.018 to − 0.003; respectively). However, no significant associations were found between serum levels of resistin and BMD at any site measured.

**Conclusions:**

Serum levels of leptin and adiponectin were significantly and negatively associated with BMD, suggesting potentially detrimental effects of leptin and adiponectin on BMD in knee OA patients.

## Background

Osteoarthritis (OA) is the most prevalent joint disease worldwide, characterized by gradual loss of articular cartilage, synovial inflammation, osteophyte formation, and other structural changes. OA affected approximately 18% of women and 10% of men aged over 60 years according to the WHO’s report [1].

Obesity is a well-recognized risk factor for OA, particularly in the weight-bearing joints. However, obesity-increased joint loading could not account for the associations between OA and non-weight-bearing joints such as hand and shoulder joints. Recent studies considered that obesity-related metabolic inflammation might contribute to OA [2, 3].

Adipokines, including leptin, adiponectin, and resistin which are mostly studied, are secreted by white adipose tissue, and have been found in synovial fluid and cartilage tissues obtained from OA patients [4, 5]. Leptin had been shown to be positively associated with the severity of OA [6, 7]; however, leptin was significantly associated with increased knee cartilage volume in patients with radiographic OA [8]. Another study [9] reported that leptin was not significantly associated with cartilage damage in OA patients. The role of adiponectin in OA remains inconclusive. Some studies suggested a protective effect of adiponectin in OA [8, 10, 11], while others found no association [12, 13] or even a positive association between serum adiponectin and disease severity in knee OA [14]. Studies regarding correlations between resistin and OA are sparse and have been controversial [15, 16].

The relationship between OA and bone mineral density (BMD) has been reported in various cross-sectional and longitudinal studies, but remains controversial. Previous studies revealed that higher BMD was associated with an increased risk of incident OA defined by osteophyte or Kellgren-Lawrence (KL) grade, suggesting that increased BMD was a risk factor for OA [17–19], but a recent study using MRI reported a positive association between systemic and subchondral BMD and cartilage thickness in patients with radiographic OA, indicating that BMD may play a protective role in OA [20].

Given that both adipokines and BMD might be involved in the etiology of OA, they would have a close relationship; however, associations between adipokines and BMD in OA are rarely reported though numerous studies reported the associations between adipokines and BMD in healthy human which remains controversial. One study was conducted in 60 postmenopausal women with hip or knee OA and reported no correlation between leptin and BMD [21]. Another study found that whole body BMD was positively correlated with the serum leptin level in 50 postmenopausal women with knee OA, but the correlation disappeared after adjustment for covariates [22]. To the best of our knowledge, there were no studies reporting the associations between adiponectin, resistin and BMD in OA patients so far. The aim of this study, therefore, was to investigate the cross-sectional associations between serum adipokines levels and BMD in patients with knee OA.

## Methods

### Subjects

This study was part of the Anhui Osteoarthritis (AHOA) Study, a clinical study of 205 patients aged 34-74 years, aimed to identify the environmental and biochemical factors associated with the progression of knee OA. Patients with clinical knee OA, diagnosed using American College of Rheumatology criteria [23], were consecutively recruited from the Department of Rheumatology and Immunology in the First Affiliated Hospital of Anhui Medical University, from January 2012 to November 2013. We excluded institutionalized patients, patients with rheumatoid arthritis or other inflammatory diseases, patients with severe OA planning to have knee arthroplasty in 2 years, patients who did not have blood samples so the adipokines were not able to be measured, and patients who didn’t have BMD measured due to personal reasons. Forty-one patients fulfilled the exclusion criteria and therefore were excluded from this study, leaving 164 patients. The study was approved by the First Affiliated Hospital Anhui Medical University ethics committee (the ethics approval number: H1000589), and written informed consent was obtained from all participants according to the Declaration of Helsinki.

### Anthropometrics

Weight was measured to the nearest 0.1 kg (with shoes, socks and bulky clothing removed) by using a single pair of electronic scales that were calibrated using a known weight at the beginning. Height was measured to the nearest 0.1 cm (with shoes, socks and headgear removed) by using a stadiometer. Body mass index (BMI) was calculated [weight (kg)/height (m)^2^].

### Serum adipokines measurements

Fasting blood was obtained from all patients in the morning. Serum was separated and aliquotted into plastic storage tubes. Aliquots were stored at − 80 °C till analysis. Serum levels of leptin, adiponectin, and resistin were measured by using enzyme-linked immunosorbent assay (ELISA; eBioscience, USA) kits, according to the manufacturer’s instructions. The intra- and inter-assay coefficient of variations for leptin, adiponectin, and resistin were 5.7 and 6.9%, 4.2 and 3.1%, 5.1 and 8.1%, respectively.

### BMD measurement

BMD of the total body, spine, hip and total femur, including femoral neck, Wards triangle, greater trochanter, and femoral shaft were measured using dual-energy x-ray absorptiometry (DXA) (Lunar Prodigy DF + 310,504, GE Healthcare, USA). BMD was calculated from the bone area (cm^2^) and bone mineral content (g) and expressed in g/cm^2^ [24]. The unit of BMD was converted to kg/m^2^ to keep the levels of adipokines and BMD at the similar magnitudes.

### Knee radiographic assessment

A standing anteroposterior semiflexed view of the diseased knee (the severer one if both were affected) with 15° of fixed knee flexion, was performed in all participants. KL grading system (grades 0-4) was used to assess the radiographic severity of OA [25]. Radiographic OA (ROA) was defined as KL grade of ≥2.

### Statistical analysis

Student’s t tests, Mann-Whitney U tests or chi-squared tests were used to compare means, median or proportions, respectively. Univariable and multivariable linear regression analyses were used to examine the associations between adipokines and BMD before and after adjustment for age, sex, BMI and ROA. Scatter plots were also used to depict the associations between adipokines and BMD after adjustment for the above-mentioned covariates. Standard diagnostic checks of model fit and residuals were routinely made, and data points with large residuals and/or high influence were investigated for data errors. A *p* value < 0.05 (two-tailed) or a 95% confidence interval (CI) not including the null point was regarded as statistically significant. All statistical analyses were performed using SPSS 13.0 for Windows (SPSS, Chicago, IL, USA).

## Results

A total of 164 subjects between 34 and 74 years of age (mean, 55.4 yrs) participated in our study. Of these, all subjects measured the BMD of total body and total femur, however, only 133 subjects measured the BMD of spine and hip. There were no significant differences in demographic factors (age, sex, and BMI) between these participants and those excluded (*n* = 41; data not shown). Characteristics of the participants are presented in Table 1. The mean BMI was 25.84 kg/m^2^. The median levels of leptin, adiponectin, and resistin were 5.92 ng/ml, 27.09μg/ml, and 2.22 ng/ml, respectively. Subjects with higher and lower levels of leptin (split at the median level) were similar in age, height, BMD at all sites measured, prevalence of ROA and levels of resistin; however, subjects with higher leptin levels had greater proportion of females, higher weight, higher BMI and higher adiponectin levels.

**Table 1:** Characteristics of participants (split by median level of leptin)

	Total (*n* = 164)	Leptin ≤ median (*n* = 82)	Leptin > median (n = 82)	*p* value
Age, yrs^a^	55.42(8.57)	54.57(8.94)	56.27(8.14)	0.206
Females, %^b^	88.4	81.7	95.1	**0.015**
Height, cm^a^	158.64(6.83)	158.62(7.70)	158.66(5.90)	0.971
Weight, kg^a^	65.07(10.29)	63.00(9.57)	67.08(10.61)	**0.012**
BMI, kg/m^2a^	25.84(3.75)	24.98(2.83)	26.68(4.32)	**0.004**
BMD, kg/m^2^				
Total body^a^	10,55(1.22)	10.64(1.41)	10.46(1.02)	0.361
Spine^a^	10.03(1.34)	10.09(1.66)	9.99(1.08)	0.709
Hip^a^	8.65(1.23)	8.90(1.42)	8.47(1.06)	0.060
Total femur^a^	9.31(1.30)	9.43(1.37)	9.21(1.24)	0.283
Knee ROA, %^b^	71.95	78.05	65.85	0.082
Adiponectin, ug/ml^c^	27.09(6.80,60.88)	11.73(3.56,35.25)	49.70(20.62,74.10)	**< 0.001**
Resistin, ng/ml^c^	2.22(1.40,4.55)	2.06(1.17,4.39)	2.27(1.48,4.63)	0.327

Leptin median level: 5.92 ng/ml

Data in bold denote statistically significant results

*BMI* body mass index, *BMD* bone mineral density, *BMC* bone mineral content, *ROA* radiographic osteoarthritis

^a^t tests were used for mean (standard deviation), ^b^x^2^ tests for the proportions, ^c^Mann-Whitney U tests for median (interquartile range)

There were no interactions between serum levels of adipokines and sex on the BMD (data not shown). Therefore, males and females were combined for analyses in our study.

**Fig. 1:**
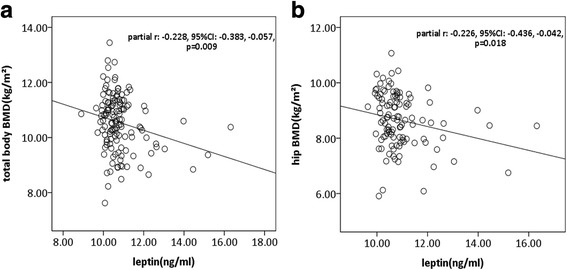
Scatter plot for associations between serum levels of leptin and bone mineral density (BMD). In multivariable analyses, higher serum levels of leptin were associated with lower BMD at total body (**a**) and hip (**b**)

Associations between leptin and BMD were shown in Table 2. In univariable analyses, we did not find any significant associations between serum levels of leptin and BMD at any site measured. However, after adjustment for age, sex, BMI and ROA, serum levels of leptin were significantly associated with reduced BMD at total body, hip, femoral neck, femoral shaft, and total femur (Table 2, Fig. 1).

**Table 2:** Associations between leptin and BMD in various regions

	Univariable		Multivariable^a^	
	β(95% CI)	*p* value	β(95% CI)	*p* value
Total body BMD	−0.011(− 0.026,0.004)	0.155	**− 0.019(− 0.034,-0.005)**	**0.009**
Spine BMD	0.004(− 0.013,0.021)	0.653	− 0.010(− 0.028,0.007)	0.248
Hip BMD	− 0.010(− 0.025,0.006)	0.213	**− 0.018(− 0.034,-0.003)**	**0.018**
Total femur BMD	− 0.007(− 0.023,0.008)	0.352	**− 0.018(− 0.034,-0.002)**	**0.024**
Femoral neck	−0.010(− 0.025,0.006)	0.218	**−0.016(− 0.032,0.000)**	**0.048**
Wards triangle	−0.007(− 0.025,0.011)	0.462	−0.012(− 0.029,0.006)	0.194
Greater trochanter	−0.004(− 0.018,0.009)	0.538	−0.013(− 0.026,0.001)	0.074
Femoral shaft	−0.008(− 0.028,0.011)	0.386	**−0.026(− 0.046,-0.006)**	**0.012**

Dependent variable: BMD in respective compartment

Independent variable: leptin

Data in bold denote statistically significant results

*BMD* bone mineral density, *BMI* body mass index, *ROA* radiographic osteoarthritis

^a^Adjusted for age, sex, BMI and ROA

**Fig. 2:**
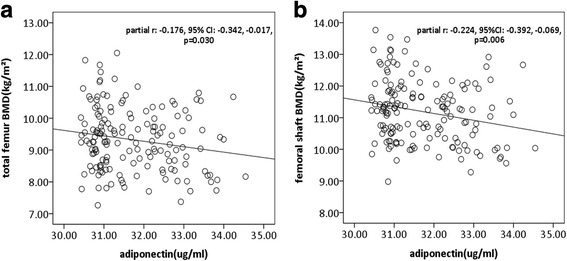
Scatter plot for associations between serum levels of adiponectin and bone mineral density (BMD). In multivariable analyses, higher serum levels of adiponectin were associated with lower BMD at total femur (**a**) and femoral shaft (**b**)

Serum adiponectin was significantly and negatively associated with BMD at femoral shaft and total femur in univariable analyses. These negative associations remained unchanged after adjustment for the covariates mentioned above (Table 3, Fig. 2). We did not find any significant associations between serum adiponectin and BMD at total body, spine, and hip. The association between adiponectin and femoral neck BMD was also negative but of borderline statistical significance (Table 3).

**Table 3:** Associations between adiponectin and BMD in various regions

	Univariable		Multivariable^a^	
	β(95% CI)	*p* value	β(95% CI)	*p* value
Total body BMD	−0.005(− 0.010,0.000)	0.072	−0.002(− 0.007,0.003)	0.432
Spine BMD	−0.006(− 0.013,0.002)	0.123	−0.002(− 0.010,0.005)	0.518
Hip BMD	−0.006(− 0.012,0.001)	0.100	−0.002(− 0.009,0.004)	0.456
Total femur BMD	**−0.006(− 0.012,0.000)**	**0.034**	**−0.007(− 0.013,0.000)**	**0.030**
Femoral neck	−0.006(− 0.011,0.000)	0.061	−0.005(− 0.011,0.001)	0.088
Wards triangle	−0.005(− 0.012,0.002)	0.137	−0.004(− 0.010,0.003)	0.234
Greater trochanter	−0.004(− 0.009,0.001)	0.116	−0.002(− 0.007,0.003)	0.436
Femoral shaft	**−0.008(− 0.015,-0.001)**	**0.025**	**−0.011(− 0.018,-0.003)**	**0.006**

Dependent variable: BMD in respective compartment

Independent variable: adiponectin

Data in bold denote statistically significant results

*BMD* bone mineral density, *BMI* body mass index, *ROA* radiographic osteoarthritis

^a^Adjusted for age, sex, BMI and ROA

The associations between serum resistin and BMD at any site did not reach statistical significance before and after adjustment for age, sex, BMI and ROA (Table 4).

**Table 4:** Associations between resistin and BMD in various regions

	Univariable		Multivariable^a^	
	β(95% CI)	*p* value	β(95% CI)	*p* value
Total body BMD	0.005(−0.042,0.053)	0.825	0.005(−0.037,0.048)	0.801
Spine BMD	−0.022(− 0.117,0.072)	0.639	− 0.024(− 0.120,0.071)	0.612
Hip BMD	− 0.005(− 0.093,0.083)	0.913	0.012(− 0.072,0.096)	0.777
Total femur BMD	− 0.010(− 0.061,0.042)	0.712	−0.007(− 0.056,0.043)	0.792
Femoral neck	−0.023(− 0.075,0.029)	0.376	−0.023(− 0.073,0.027)	0.358
Wards triangle	−0.030(− 0.090,0.030)	0.327	−0.031(− 0.086,0.023)	0.257
Greater trochanter	−0.014(− 0.059,0.031)	0.528	−0.012(− 0.054,0.030)	0.568
Femoral shaft	0.002(−0.062,0.066)	0.951	0.003(−0.061,0.067)	0.918

Dependent variable: BMD in respective compartment

Independent variable: resistin

*BMD* bone mineral density, *BMI* body mass index, *ROA* radiographic osteoarthritis

^a^Adjusted for age, sex, BMI and ROA

## Discussion

The present study investigated the cross-sectional associations between adipokines (including leptin, adiponectin, and resistin) and BMD in patients with knee OA. We found that the serum leptin levels were negatively associated with total body, hip and total femur (including femoral neck and femoral shaft) BMD. Serum adiponectin was also significantly associated with reduced BMD at total femur and femoral shaft. In contrast, no association was found between serum resistin and BMD.

Leptin, a protein encoded by the ob gene, was found to regulate bone metabolism. Various studies have demonstrated the association between leptin and BMD. Ducy et al. [26] found that intracerebroventricular infusion of leptin induced bone loss in both leptin-deficient and wild-type mice, suggesting that leptin could inhibit bone formation acting through the hypothalamus. Epidemiological studies reported that serum leptin concentrations were inversely associated with calcaneal BMD after adjustment for body weight in 221 healthy adult men [27], and leptin had a negative correlation with lumbar spine BMD in perimenopausal healthy women [28]. On the contrary, some studies reported a positive association between leptin and BMD. Martin et al. [29] demonstrated that peripheral administration of leptin could prevent disuse-induced bone loss through inhibiting the increase in bone resorption mediated by the RANKL/OPG and preventing the decrease in bone formation in tail-suspended female rats. Blain et al. [30] found that leptin was positively associated with whole body and femoral neck BMD in 155 postmenopausal women. Weiss et al. [31] reported that leptin predicted an increase in BMD in postmenopausal women but not older men after adjustment for age, BMI, and other bone related factors. In addition, several clinical studies reported no association between leptin and BMD or BMD change [32, 33]. It was noteworthy that these studies mentioned above were conducted in healthy persons. Only two clinical studies investigated the association between leptin and BMD in OA [21, 22] and reported inconsistent results. Our study found that serum leptin was significantly associated with reduced BMD in patients with knee OA, independent of age, sex, BMI, and ROA. This suggests that leptin may play a potentially detrimental effect on BMD in knee OA.

Adiponectin was considered to have a negative effect on bone metabolism. Adiponectin levels were negatively associated with femoral neck and total body BMD in postmenopausal women after adjustment for potential confounders [32], and highest tertile of adiponectin had significantly greater hip BMD loss than the lowest tertile of adiponectin in women [33]. A meta-analysis indicated that adiponectin was the mostly relevant adipokine that was negatively associated with BMD in healthy subjects, regardless of menopausal status and gender [34]. Oshima et al. [35] reported that adiponectin supplement increased bone mass in trabecular bone via inhibiting osteoclast and activating osteoblast. In contrast, Kontogianni et al. [28] found no associations between adiponectin and lumber spine BMD in perimenopausal women, and Barbour et al. [33] found adiponectin levels were not correlated with whole-body areal BMD or trabecular lumbar spine volumetric BMD loss in older women and men. Our current study is the first to investigate the association between adiponectin and BMD in patients with knee OA, indicating a potentially detrimental effect of adiponectin on BMD in knee OA.

Serum leptin levels are positively correlated with BMI, while serum adiponectin levels are negatively correlated with BMI [36]. However, we found that both serum levels of leptin and adiponectin were negatively associated with BMD in OA. The potential mechanism is unclear, and it needs to be further investigated.

So far, there are a few studies reporting the association between resistin and BMD. Serum resistin was not associated with BMD of total body, lumbar spine, and total hip before and after adjustment for age and fat mass in 232 Chinese men [37], and was not an independent predictor of BMD in 336 healthy postmenopausal Chinese women aged 41-81 years [38]. One study reported that serum resistin levels were significantly and negatively associated with lumbar spine BMD in 80 middle-aged men [39]. In our current study, we didn’t find any significant association between serum resistin and BMD in patients with knee OA.

There are several limitations in our study. First, due to the cross-sectional nature, the causality between adipokines and BMD is not able to be determined. Further longitudinal studies are needed to verify our findings. Second, the sample size was modest. This may be the reason why we did not find significant association between resistin and BMD. Third, 41 patients were excluded from this study which may cause selection bias; however, there were no significant differences in age, sex, and BMI between patients who were included and excluded. Last, there would be variations in muscle and fat mass which may affect the associations; however, the results remained largely unchanged after the adjustment for muscle or fat mass.

## Conclusions

Serum leptin and adiponectin levels were significantly and negatively associated with BMD, suggesting potentially detrimental effects of leptin and adiponectin on BMD in knee OA patients.

## Abbreviations

BMD: Bone mineral density
BMI: Body mass index
CI: Confidence interval
DXA: Dual-energy X-ray absorptiometry
ELISA: Enzyme-linked immunosorbent assay
KL: Kellgren-Lawrence
OA: Osteoarthritis
ROA: Radiographic OA
